# A Brief Retrospect of Matters Surgical

**Published:** 1891-05

**Authors:** T. Dwight Stow

**Affiliations:** Mexico, New York; Bureau of Surgery, I. H. A.


### A BRIEF RETROSPECT OF MATTERS SURGICAL.

T. Dwight Stow, M. D., Mexico, New York.

(Bureau of Surgery, I. H. A.)

Fellow-Members I. H. A. :—One year ago our highly respected colleague, Dr. James B. Bell, of Boston, then chairman of the Bureau of Surgery, treated us to a rare, comprehensive, and very instructive paper on "Listerism," a copy of which should be in the library of every physician and surgeon. Since the publication of that paper, I have looked in vain, in such old-school papers and journals as have been in my reach, for some review or criticism of the same, but, probably because I take a limited number of such journals, have not seen any notice or review of the paper. But all unintentionally, the trend of old-school surgical therapeia has been in the direction of asepsis and away from what is styled antisepsis, a marked tribute to Dr. Bell's timely and truthful brochure as well as to the Doctor's forethought in producing it. "Listerism," so far as its so-called antiseptic agents are concerned, seems to be seriously imperiled among its own, and the old quotation, "He came to His own, and His own received Him not," is now in process of verification, for, one by one, antisepsis is being abandoned by those who first advocated it.

On page 1 of the *International Journal of Surgery* for January, 1890, in summing up surgical progress in 1889, we find:

"The value of Iodoform as an antiseptic has been found less than was at first thought to be the case, but its efficacy in the treatment of tubercular affections is now better recognized, and good results have followed its injection in tubercular joints."

Again:

"The toxic effects brought about by the absorption of Corrosive sublimate, in the employment of this substance as an antiseptic agent within the large cavities of the body, have led to a gradual abandonment of this drug for that purpose. It is now principally replaced by the use of water sterilized by boiling."

On page 48 of the same journal, and under the heading, "The Influence of Ventilation upon Micro-organisms Suspended in the Air," we further find: "And sprays are also worthless for the purpose of disinfection of the air." Quotations like the above might be indefinitely multiplied, but as it is my purpose to give but a *résumé* of the status of surgery at the present time, I forbear. It is evident that the disposition of chemists and pharmacists is to continually discover and combine and give new names to new agents and combinations; and equally the tendency of surgeons to make corresponding and radical changes in their antiseptic treatment, thus clouding them with a well-founded suspicion of unreliability. Carbolic acid, Corrosive sublimate, Iodoform, Acetic acid, etc., are being replaced by the free use of pure water as an irrigant, the sterilization of instruments, hands, towels, dressings, by heat, dry and moist, free drainage, and so on. It is true that many surgeons, who have abandoned the now fading "Listerism" of the near past, seize with their first avidity new antiseptics such as "Aristol," "Campho," "Phenique" "Sanitas," "Hydronaphthol," etc., but these will in time share the fate of obsolete and dying antiseptics. Sir Joseph Lister, even, questioning the value of his "double Cyanide of Zinc and Mercury *with Starch*," has accordingly ordered his manufacturing chemists to stop its production. (See *International Journal of Surgery*, Vol. III, No. 1, page 24.) Probably the safest, best aseptics known today are Hydronaphthol and Lloyd's Asepsin. The attention of the profession is being fixed upon these and some coming germicide, to be drawn from the Phenol group.

In the field of *operative* surgery, well-marked advancement has been made. In abdominal surgery, an improved technique; earlier operations, operations even during the presence of peritonitis; the large abandonment of Opium; the treatment of "appendicitis" by operation; the treatment of perityphlitic abscess; improvements in the treatment of anastomotic operations on stomach and intestines, are sure evidences of the strides taken.

Topographical studies of *cerebral lesions*, and the almost mathematical accuracy of operations therefor, by trephining, the removal of tumors, the evacuation of abscesses, the relief of compression of the cord by removing fragments of bone; the surgery of the nerves, such as resection of nerves for the treatment of neuralgias; suture of divided nerve-ends; improved operations on the bladder, prostate gland, and the substitution of supra-pubic operations for perineal section, and Professor Bellfield's radical "prostatectomy" are most conspicuous. All in all, surgery has kept abreast of the age. Indeed, it has gone ahead!

### Venereal Diseases.

I desire to present a few thoughts in respect to the present classification of syphilis, whether primary, secondary, or tertiary, and gonorrhœa.

At some time in the remote past, probably because at such time the treatment of ulcers and other external—or for that matter—internal manifestations was by the use of the knife, cautery, lotions, ointments requiring more or less *manipulation*, the treatment of patients suffering from syphilis, gonorrhœa, sycosis was turned over to the surgeon in whose hands mainly we find it to-day. To this mere fact I have no objection: for it must be admitted that a surgeon may lay down his knife, ligature, and cautery, and treat such cases in accordance with general theory as in the old school; or in accordance with *law*, as in our own—and be successful, too—*but in this case he becomes a therapeutician.* What I wish to know is: Why the diagnosis, prognosis, and treatment of venereal disease, and papers relating to the same, should be consigned to the Bureau of Surgery? Are the diseases in view so truly local, so amenable to operative attention, or so foul as to make such consignment inevitable and necessary? Orthographically and etymologically considered, surgery is that branch of the healing art that pertains to Xeipos, hand, and Epyon, work. It is that branch of the healing art that teaches the proper use of *manual* operations for the preservation or restoration of health, including the exhibition of such medicinal agents as will facilitate the reparation of lesions or cure of morbid growths.

These thoughts are not offered in a captious or critical mood, but to suggest that syphilis, sycosis, gonorrhœa belong as much and *more* to the departments or Bureau of Clinical Medicine and Materia Medica, than to the Bureau of Surgery. "*Laus illis guibas debentur.*"

Not in ninety cases of a hundred, but in *all* cases, the *cure*, the *radical* cure of venereal disease is effected by constitutional, general systemic treatment: *by strict therapeutic and hygienic processes,* hence outside the realm of surgery. Therefore, I offer the following motion:

Moved: That, in the future, so far as the interests of science and this Association are concerned—recognizing the natural order and fitness of things—all articles relating to the therapeutics of "Venereal diseases" be referred to the Bureau of Clinical Medicine—excepting such cases as require strictly surgical interference in conjunction with remedies, homœopathic to them.

